# 3-[2-Hy­droxy-3-(2,4,6-trimethyl­phen­yl)prop­yl]-3-methyl-1-phenyl­thio­urea

**DOI:** 10.1107/S1600536811012736

**Published:** 2011-04-13

**Authors:** Abel M. Maharramov, Ali N. Khalilov, Nurlana D. Sadikhova, Atash V. Gurbanov, Seik Weng Ng

**Affiliations:** a Department of Organic Chemistry, Baku State University, Baku, Azerbaijan; b Department of Chemistry, University of Malaya, 50603 Kuala Lumpur, Malaysia

## Abstract

In the title compound, C_20_H_26_N_2_OS, four non-H atoms of the thio­urea unit are approximately planar (r.m.s. deviation = 0.005 Å); the phenyl and benzene rings are twisted out of this plane by 28.55 (7) and 60.00 (7)°, respectively. An intra­molecular N—H⋯O hydrogen bond occurs. The hy­droxy group is hydrogen bonded to the double-bond S atom of an inversion-related mol­ecule, generating a hydrogen-bonded dimer in the crystal structure.

## Related literature

The title compund was prepared by a reaction of 1-methyl­amino-3-(2,4,6-trimethyl­phen­yl)propan-2-ol and phenyl iso­thio­cyanate; for the structure of the reactant 1-methyl­amino-3-(2,4,6-trimethyl­phen­yl)propan-2-ol, see: Maharramov *et al.* (2011 ▶).
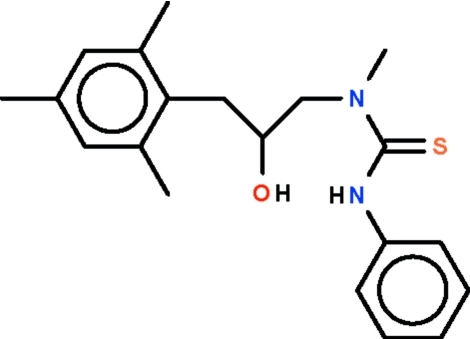


## Experimental

### 

#### Crystal data

C_20_H_26_N_2_OS
*M* *_r_* = 342.49
Monoclinic, 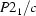

*a* = 14.6313 (11) Å
*b* = 8.1579 (6) Å
*c* = 16.4455 (12) Å
β = 109.040 (1)°
*V* = 1855.6 (2) Å^3^
*Z* = 4
Mo *K*α radiation
μ = 0.18 mm^−1^
*T* = 100 K
0.30 × 0.20 × 0.20 mm

#### Data collection

Bruker SMART APEX diffractometer
Absorption correction: multi-scan (*SADABS*; Sheldrick, 1996 ▶) *T*_min_ = 0.947, *T*_max_ = 0.964
10052 measured reflections
4160 independent reflections
3542 reflections with *I* > 2σ(*I*)
*R*_int_ = 0.028

#### Refinement

*R*[*F* ^2^ > 2σ(*F* ^2^)] = 0.039
*wR*(*F* ^2^) = 0.112
*S* = 1.04
4160 reflections
229 parameters
2 restraints
H atoms treated by a mixture of independent and constrained refinement
Δρ_max_ = 0.38 e Å^−3^
Δρ_min_ = −0.24 e Å^−3^

### 

Data collection: *APEX2* (Bruker, 2005 ▶); cell refinement: *SAINT* (Bruker, 2005 ▶); data reduction: *SAINT*; program(s) used to solve structure: *SHELXS97* (Sheldrick, 2008 ▶); program(s) used to refine structure: *SHELXL97* (Sheldrick, 2008 ▶); molecular graphics: *X-SEED* (Barbour, 2001 ▶); software used to prepare material for publication: *publCIF* (Westrip, 2010 ▶).

## Supplementary Material

Crystal structure: contains datablocks global, I. DOI: 10.1107/S1600536811012736/xu5185sup1.cif

Structure factors: contains datablocks I. DOI: 10.1107/S1600536811012736/xu5185Isup2.hkl

Additional supplementary materials:  crystallographic information; 3D view; checkCIF report

## Figures and Tables

**Table 1:** Hydrogen-bond geometry (Å, °)

*D*—H⋯*A*	*D*—H	H⋯*A*	*D*⋯*A*	*D*—H⋯*A*
O1—H1⋯S1^i^	0.83 (1)	2.50 (1)	3.219 (1)	146 (2)
N2—H2⋯O1	0.88 (1)	1.89 (1)	2.739 (2)	165 (2)

Symmetry code: (i) 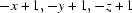.

**Fig. 1.**
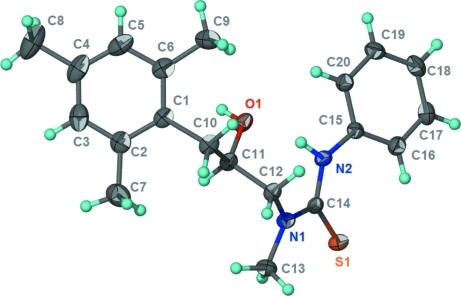
Thermal ellipsoid plot (Barbour, 2001) of C20H26N2OS at the 70% probability level; hydrogen atoms are drawn as spheres of arbitrary radius.

## supplementary crystallographic information

### Comment

We have recently reported the synthesis and crystal structure of 1-methylamino-3-(2,4,6-trimethylphenyl)propan-2-ol (Maharramov *et al.*, 2011). This secondary amine behaves like a conventional secondary amine in its reaction with phenyl isothiocyanate to furnish a thiourea (Scheme I). The four-atoms N–C(═S)–N unit of C_20_H_26_N_2_OS is planar [r.m.s. deviation 0.005 Å]; the phenyl ring connected to one of the two flanking N atoms is twisted out of this plane 28.6 (1)° (Fig. 1). The propyl chain connected to the other N atom bears a hydroxy substituent; this serves as hydrogen-bond donor acceptor to the double-bond S atom of an inversion-related molecule to generate a hydrogen-bonded dimer.

### Experimental

1-Methylamino-3-(2,4,6-trimethylphenyl)propan-2-ol was synthesized as reported (Maharramov *et al.*, 2011). The compound (10 mmol) and phenyl isothiocyanate (10 mmol) were heated in benzene (50 mol) for 10 h. The solvent was removed and the product recrystallized from ethanol to yield colorless crystals, m.p. 413–414 K; yield 90%.

### Refinement

Carbon-bound H-atoms were placed in calculated positions [C–H 0.95 to 1.00 Å; *U*(H) 1.2 to 1.5*U*(C)] and were included in the refinement in the riding model approximation.

The hydroxy and amino H-atoms were located in a difference Fourier map, and were refined with distance restraints of O–H 0.84±0.01 and N–H 0.88±0.01 Å; their temperature factors were refined.

### Crystal data

**Table:**

C_20_H_26_N_2_OS	*F*(000) = 736
*M**_r_* = 342.49	*D*_x_ = 1.226 Mg m^−^^3^
Monoclinic, *P*2_1_/*c*	Mo *K*α radiation, λ = 0.71073 Å
Hall symbol: -P 2ybc	Cell parameters from 4968 reflections
*a* = 14.6313 (11) Å	θ = 2.6–29.2°
*b* = 8.1579 (6) Å	µ = 0.18 mm^−^^1^
*c* = 16.4455 (12) Å	*T* = 100 K
β = 109.040 (1)°	Prism, colorless
*V* = 1855.6 (2) Å^3^	0.30 × 0.20 × 0.20 mm
*Z* = 4	

### Data collection

**Table:**

Bruker SMART APEX diffractometer	4160 independent reflections
Radiation source: fine-focus sealed tube	3542 reflections with *I* > 2σ(*I*)
graphite	*R*_int_ = 0.028
φ and ω scans	θ_max_ = 27.5°, θ_min_ = 2.6°
Absorption correction: multi-scan (*SADABS*; Sheldrick, 1996)	*h* = −18→18
*T*_min_ = 0.947, *T*_max_ = 0.964	*k* = −10→10
10052 measured reflections	*l* = −20→20

### Refinement

**Table:**

Refinement on *F*^2^	Primary atom site location: structure-invariant direct methods
Least-squares matrix: full	Secondary atom site location: difference Fourier map
*R*[*F*^2^ > 2σ(*F*^2^)] = 0.039	Hydrogen site location: inferred from neighbouring sites
*wR*(*F*^2^) = 0.112	H atoms treated by a mixture of independent and constrained refinement
*S* = 1.04	*w* = 1/[σ^2^(*F*_o_^2^) + (0.0617*P*)^2^ + 0.4837*P*] where *P* = (*F*_o_^2^ + 2*F*_c_^2^)/3
4160 reflections	(Δ/σ)_max_ = 0.001
229 parameters	Δρ_max_ = 0.38 e Å^−^^3^
2 restraints	Δρ_min_ = −0.24 e Å^−^^3^

### Fractional atomic coordinates and isotropic or equivalent isotropic displacement parameters (Å^2^)

**Table:**

	*x*	*y*	*z*	*U*_iso_*/*U*_eq_	
S1	0.35275 (3)	0.49063 (4)	0.61941 (2)	0.02172 (11)	
O1	0.61082 (7)	0.26588 (12)	0.52099 (7)	0.0223 (2)	
H1	0.6434 (13)	0.330 (2)	0.5027 (12)	0.041 (6)*	
N1	0.51088 (8)	0.30365 (14)	0.65553 (7)	0.0197 (3)	
N2	0.42327 (8)	0.31882 (14)	0.51317 (8)	0.0196 (2)	
H2	0.4785 (9)	0.292 (2)	0.5069 (12)	0.032 (5)*	
C1	0.81508 (10)	0.13935 (16)	0.59751 (10)	0.0216 (3)	
C2	0.89199 (11)	0.21843 (18)	0.65953 (10)	0.0260 (3)	
C3	0.97052 (11)	0.2735 (2)	0.63719 (12)	0.0320 (4)	
H3	1.0215	0.3293	0.6790	0.038*	
C4	0.97654 (12)	0.2495 (2)	0.55560 (12)	0.0340 (4)	
C5	0.90094 (12)	0.1692 (2)	0.49568 (11)	0.0319 (4)	
H5	0.9043	0.1508	0.4397	0.038*	
C6	0.82014 (11)	0.11434 (18)	0.51461 (10)	0.0254 (3)	
C7	0.89290 (13)	0.2418 (2)	0.75097 (11)	0.0364 (4)	
H7A	0.9484	0.3098	0.7824	0.055*	
H7B	0.8979	0.1348	0.7792	0.055*	
H7C	0.8330	0.2961	0.7506	0.055*	
C8	1.06343 (14)	0.3063 (2)	0.53256 (16)	0.0496 (5)	
H8A	1.0423	0.3813	0.4834	0.074*	
H8B	1.0956	0.2112	0.5174	0.074*	
H8C	1.1087	0.3630	0.5819	0.074*	
C9	0.74118 (12)	0.0268 (2)	0.44548 (11)	0.0327 (4)	
H9A	0.7578	0.0233	0.3924	0.049*	
H9B	0.6800	0.0856	0.4348	0.049*	
H9C	0.7345	−0.0852	0.4643	0.049*	
C10	0.72773 (10)	0.08517 (17)	0.62059 (10)	0.0223 (3)	
H10A	0.7491	0.0465	0.6810	0.027*	
H10B	0.6964	−0.0080	0.5834	0.027*	
C11	0.65378 (10)	0.22347 (17)	0.61007 (9)	0.0195 (3)	
H11	0.6861	0.3218	0.6435	0.023*	
C12	0.57030 (10)	0.16888 (16)	0.64028 (9)	0.0195 (3)	
H12A	0.5280	0.0943	0.5965	0.023*	
H12B	0.5971	0.1057	0.6942	0.023*	
C13	0.54069 (11)	0.36552 (19)	0.74324 (9)	0.0253 (3)	
H13A	0.5239	0.4819	0.7427	0.038*	
H13B	0.6107	0.3523	0.7697	0.038*	
H13C	0.5075	0.3040	0.7765	0.038*	
C14	0.43142 (10)	0.36483 (16)	0.59459 (9)	0.0175 (3)	
C15	0.34745 (10)	0.33743 (15)	0.43473 (9)	0.0177 (3)	
C16	0.25039 (10)	0.36157 (17)	0.42568 (10)	0.0228 (3)	
H16	0.2307	0.3745	0.4749	0.027*	
C17	0.18274 (11)	0.36650 (18)	0.34365 (10)	0.0274 (3)	
H17	0.1166	0.3841	0.3374	0.033*	
C18	0.20929 (11)	0.34643 (19)	0.27106 (10)	0.0280 (3)	
H18	0.1619	0.3497	0.2155	0.034*	
C19	0.30580 (11)	0.32148 (18)	0.28011 (9)	0.0251 (3)	
H19	0.3249	0.3065	0.2307	0.030*	
C20	0.37432 (10)	0.31839 (16)	0.36127 (9)	0.0212 (3)	
H20	0.4405	0.3031	0.3671	0.025*	

### Atomic displacement parameters (Å^2^)

**Table:**

	*U*^11^	*U*^22^	*U*^33^	*U*^12^	*U*^13^	*U*^23^
S1	0.0242 (2)	0.02255 (18)	0.0209 (2)	0.00347 (13)	0.01083 (14)	0.00010 (13)
O1	0.0211 (5)	0.0255 (5)	0.0227 (5)	0.0022 (4)	0.0102 (4)	0.0086 (4)
N1	0.0209 (6)	0.0219 (6)	0.0169 (6)	0.0021 (4)	0.0072 (5)	0.0012 (4)
N2	0.0176 (6)	0.0247 (6)	0.0176 (6)	0.0025 (5)	0.0074 (5)	0.0004 (5)
C1	0.0210 (7)	0.0185 (6)	0.0264 (7)	0.0061 (5)	0.0092 (6)	0.0057 (5)
C2	0.0215 (7)	0.0253 (7)	0.0294 (8)	0.0078 (6)	0.0056 (6)	0.0039 (6)
C3	0.0195 (7)	0.0279 (8)	0.0450 (10)	0.0049 (6)	0.0057 (7)	0.0036 (7)
C4	0.0255 (8)	0.0285 (8)	0.0531 (11)	0.0098 (6)	0.0196 (7)	0.0149 (7)
C5	0.0342 (9)	0.0333 (8)	0.0348 (9)	0.0116 (7)	0.0203 (7)	0.0108 (7)
C6	0.0267 (8)	0.0221 (7)	0.0285 (8)	0.0090 (6)	0.0105 (6)	0.0062 (6)
C7	0.0317 (9)	0.0433 (10)	0.0289 (9)	0.0076 (7)	0.0029 (7)	−0.0013 (7)
C8	0.0328 (10)	0.0438 (11)	0.0815 (16)	0.0091 (8)	0.0315 (10)	0.0224 (10)
C9	0.0361 (9)	0.0370 (9)	0.0247 (8)	0.0071 (7)	0.0096 (7)	−0.0018 (7)
C10	0.0236 (7)	0.0190 (6)	0.0257 (7)	0.0040 (5)	0.0101 (6)	0.0064 (5)
C11	0.0198 (7)	0.0193 (6)	0.0201 (7)	0.0017 (5)	0.0074 (5)	0.0045 (5)
C12	0.0197 (7)	0.0186 (6)	0.0211 (7)	0.0013 (5)	0.0080 (5)	0.0039 (5)
C13	0.0280 (8)	0.0306 (8)	0.0167 (7)	−0.0006 (6)	0.0064 (6)	−0.0002 (6)
C14	0.0192 (7)	0.0162 (6)	0.0188 (7)	−0.0029 (5)	0.0086 (5)	0.0009 (5)
C15	0.0200 (7)	0.0148 (6)	0.0183 (7)	−0.0014 (5)	0.0061 (5)	−0.0005 (5)
C16	0.0215 (7)	0.0233 (7)	0.0246 (7)	−0.0015 (5)	0.0089 (6)	−0.0005 (6)
C17	0.0185 (7)	0.0290 (8)	0.0315 (8)	−0.0016 (6)	0.0039 (6)	0.0005 (6)
C18	0.0272 (8)	0.0293 (8)	0.0218 (8)	−0.0048 (6)	0.0000 (6)	0.0012 (6)
C19	0.0311 (8)	0.0259 (7)	0.0177 (7)	−0.0043 (6)	0.0070 (6)	−0.0006 (6)
C20	0.0220 (7)	0.0207 (6)	0.0220 (7)	−0.0019 (5)	0.0086 (6)	−0.0011 (5)

### Geometric parameters (Å, °)

**Table:**

S1—C14	1.6885 (14)	C8—H8C	0.9800
O1—C11	1.4353 (17)	C9—H9A	0.9800
O1—H1	0.826 (9)	C9—H9B	0.9800
N1—C14	1.3583 (17)	C9—H9C	0.9800
N1—C13	1.4544 (18)	C10—C11	1.5333 (19)
N1—C12	1.4730 (17)	C10—H10A	0.9900
N2—C14	1.3578 (18)	C10—H10B	0.9900
N2—C15	1.4086 (17)	C11—C12	1.5275 (19)
N2—H2	0.875 (9)	C11—H11	1.0000
C1—C6	1.404 (2)	C12—H12A	0.9900
C1—C2	1.405 (2)	C12—H12B	0.9900
C1—C10	1.5130 (19)	C13—H13A	0.9800
C2—C3	1.390 (2)	C13—H13B	0.9800
C2—C7	1.512 (2)	C13—H13C	0.9800
C3—C4	1.387 (3)	C15—C16	1.393 (2)
C3—H3	0.9500	C15—C20	1.396 (2)
C4—C5	1.383 (3)	C16—C17	1.389 (2)
C4—C8	1.513 (2)	C16—H16	0.9500
C5—C6	1.391 (2)	C17—C18	1.380 (2)
C5—H5	0.9500	C17—H17	0.9500
C6—C9	1.510 (2)	C18—C19	1.386 (2)
C7—H7A	0.9800	C18—H18	0.9500
C7—H7B	0.9800	C19—C20	1.3835 (19)
C7—H7C	0.9800	C19—H19	0.9500
C8—H8A	0.9800	C20—H20	0.9500
C8—H8B	0.9800		
			
C11—O1—H1	114.3 (14)	C1—C10—H10A	109.1
C14—N1—C13	120.79 (12)	C11—C10—H10A	109.1
C14—N1—C12	124.00 (12)	C1—C10—H10B	109.1
C13—N1—C12	115.16 (11)	C11—C10—H10B	109.1
C14—N2—C15	131.51 (12)	H10A—C10—H10B	107.9
C14—N2—H2	113.4 (12)	O1—C11—C12	105.77 (11)
C15—N2—H2	113.7 (12)	O1—C11—C10	110.52 (11)
C6—C1—C2	119.22 (14)	C12—C11—C10	111.01 (11)
C6—C1—C10	121.17 (13)	O1—C11—H11	109.8
C2—C1—C10	119.61 (13)	C12—C11—H11	109.8
C3—C2—C1	119.47 (15)	C10—C11—H11	109.8
C3—C2—C7	118.83 (15)	N1—C12—C11	114.64 (11)
C1—C2—C7	121.68 (14)	N1—C12—H12A	108.6
C4—C3—C2	121.91 (16)	C11—C12—H12A	108.6
C4—C3—H3	119.0	N1—C12—H12B	108.6
C2—C3—H3	119.0	C11—C12—H12B	108.6
C5—C4—C3	117.90 (15)	H12A—C12—H12B	107.6
C5—C4—C8	120.58 (17)	N1—C13—H13A	109.5
C3—C4—C8	121.51 (18)	N1—C13—H13B	109.5
C4—C5—C6	122.24 (16)	H13A—C13—H13B	109.5
C4—C5—H5	118.9	N1—C13—H13C	109.5
C6—C5—H5	118.9	H13A—C13—H13C	109.5
C5—C6—C1	119.24 (15)	H13B—C13—H13C	109.5
C5—C6—C9	118.75 (15)	N2—C14—N1	113.87 (12)
C1—C6—C9	122.01 (14)	N2—C14—S1	123.98 (10)
C2—C7—H7A	109.5	N1—C14—S1	122.12 (10)
C2—C7—H7B	109.5	C16—C15—C20	119.25 (13)
H7A—C7—H7B	109.5	C16—C15—N2	125.74 (13)
C2—C7—H7C	109.5	C20—C15—N2	114.87 (12)
H7A—C7—H7C	109.5	C17—C16—C15	119.10 (14)
H7B—C7—H7C	109.5	C17—C16—H16	120.4
C4—C8—H8A	109.5	C15—C16—H16	120.4
C4—C8—H8B	109.5	C18—C17—C16	121.61 (14)
H8A—C8—H8B	109.5	C18—C17—H17	119.2
C4—C8—H8C	109.5	C16—C17—H17	119.2
H8A—C8—H8C	109.5	C17—C18—C19	119.26 (14)
H8B—C8—H8C	109.5	C17—C18—H18	120.4
C6—C9—H9A	109.5	C19—C18—H18	120.4
C6—C9—H9B	109.5	C20—C19—C18	119.91 (14)
H9A—C9—H9B	109.5	C20—C19—H19	120.0
C6—C9—H9C	109.5	C18—C19—H19	120.0
H9A—C9—H9C	109.5	C19—C20—C15	120.85 (13)
H9B—C9—H9C	109.5	C19—C20—H20	119.6
C1—C10—C11	112.30 (11)	C15—C20—H20	119.6
			
C6—C1—C2—C3	1.4 (2)	C14—N1—C12—C11	90.19 (16)
C10—C1—C2—C3	−177.61 (13)	C13—N1—C12—C11	−92.18 (14)
C6—C1—C2—C7	−177.09 (13)	O1—C11—C12—N1	−76.40 (14)
C10—C1—C2—C7	3.9 (2)	C10—C11—C12—N1	163.70 (12)
C1—C2—C3—C4	−1.5 (2)	C15—N2—C14—N1	170.80 (13)
C7—C2—C3—C4	177.01 (14)	C15—N2—C14—S1	−10.8 (2)
C2—C3—C4—C5	0.5 (2)	C13—N1—C14—N2	167.94 (12)
C2—C3—C4—C8	−178.50 (15)	C12—N1—C14—N2	−14.56 (18)
C3—C4—C5—C6	0.6 (2)	C13—N1—C14—S1	−10.48 (18)
C8—C4—C5—C6	179.65 (15)	C12—N1—C14—S1	167.02 (10)
C4—C5—C6—C1	−0.7 (2)	C14—N2—C15—C16	−22.3 (2)
C4—C5—C6—C9	−179.63 (14)	C14—N2—C15—C20	162.14 (13)
C2—C1—C6—C5	−0.3 (2)	C20—C15—C16—C17	−0.2 (2)
C10—C1—C6—C5	178.68 (13)	N2—C15—C16—C17	−175.56 (13)
C2—C1—C6—C9	178.57 (13)	C15—C16—C17—C18	0.7 (2)
C10—C1—C6—C9	−2.4 (2)	C16—C17—C18—C19	−0.3 (2)
C6—C1—C10—C11	−94.33 (16)	C17—C18—C19—C20	−0.6 (2)
C2—C1—C10—C11	84.66 (16)	C18—C19—C20—C15	1.1 (2)
C1—C10—C11—O1	67.26 (15)	C16—C15—C20—C19	−0.7 (2)
C1—C10—C11—C12	−175.70 (12)	N2—C15—C20—C19	175.19 (12)

### Hydrogen-bond geometry (Å, °)

**Table:**

*D*—H···*A*	*D*—H	H···*A*	*D*···*A*	*D*—H···*A*
O1—H1···S1^i^	0.83 (1)	2.50 (1)	3.219 (1)	146 (2)
N2—H2···O1	0.88 (1)	1.89 (1)	2.739 (2)	165 (2)

Symmetry codes: (i) −*x*+1, −*y*+1, −*z*+1.

## References

[bb1] Barbour, L. J. (2001). *J. Supramol. Chem.* **1**, 189–191.

[bb2] Bruker (2005). *APEX2* and *SAINT* Bruker AXS Inc., Madison, Wisconsin, USA.

[bb3] Maharramov, A. M., Khalilov, A. N., Gurbanov, A. V., Allahverdiyev, M. A. & Ng, S. W. (2011). *Acta Cryst.* E**67**, o784. 10.1107/S1600536811007513 PMC3099974 21754075

[bb4] Sheldrick, G. M. (1996). *SADABS* University of Göttingen, Germany.

[bb5] Sheldrick, G. M. (2008). *Acta Cryst.* A**64**, 112–122. 10.1107/S0108767307043930 18156677

[bb6] Westrip, S. P. (2010). *J. Appl. Cryst.* **43**, 920–925.

